# Variants in UGT1A1 and SLCO1B1 increase the risk of neonatal hyperbilirubinemia: a case-control study in subtropical China

**DOI:** 10.3389/fped.2026.1843414

**Published:** 2026-06-24

**Authors:** Wen-Fang Wang, Li-Sen Shi, Zeng-Wen Xing, Yan-Mei Han, Wei-Qiang Liu

**Affiliations:** 1 Clinical Laboratory Diagnostics, Shantou University Medical College, Shantou, Guangdong Province, China; 2 Medical Genetics and Prenatal Diagnosis, Haikou Maternal and Child Health Hospital, Haikou, Hainan Province, China; 3 Central Laboratory (Genetics Lab), Longgang District Maternity & Child Healthcare Hospital of Shenzhen City (Affiliated Shenzhen Women and Children's Hospital (Longgang) of Shantou University Medical College), Shenzhen, China

**Keywords:** cumulative effect, genetic susceptibility, neonatal hyperbilirubinemia, single nucleotide polymorphism, SLCO1B1, subtropical China, UGT1A1

## Abstract

**Objective:**

The genetic architecture underlying susceptibility to neonatal hyperbilirubinemia in specific subtropical populations remains poorly defined. This study aimed to evaluate the association between polymorphisms in the uridine diphosphate glucuronosyltransferase 1A1(UGT1A1) and solute carrier organic anion transporter family member 1B1 (SLCO1B1) genes and the risk of neonatal hyperbilirubinemia within the Haikou population, and to explore potential cumulative genetic effects.

**Methods:**

A retrospective case-control study was conducted involving 210 neonates diagnosed with hyperbilirubinemia (case group) and 195 healthy neonates (control group) admitted to the Haikou Maternal and Child Health Hospital between January 2024 and January 2025. Serum levels of total bilirubin (TBIL), indirect bilirubin (IBIL),and alkaline phosphatase (ALP) were quantified. Seven target single nucleotide polymorphisms (SNPs) were genotyped, including four UGT1A1 loci (rs4148323, rs3771341, rs34946978, rs35350960) and three SLCO1B1 loci (rs4149056, rs2306283, rs4149015). Genotype-phenotype correlations, disease risk associations,gene-gene interactions, cumulative risk allele analyses, and combined genotype analyses were statistically evaluated. Stratified and sensitivity analyses were performed to assess the robustness of the findings.

**Results:**

The case group exhibited a significantly higher prevalence of preterm births compared to the control group (16.9% vs. 9.5%, *P* = 0.04). Allele frequencies for UGT1A1 rs4148323 (22.14% vs. 11.79%), SLCO1B1 rs4149056 (19.05% vs. 10.26%), and rs4149015 (21.43% vs. 11.79%) were significantly elevated in the case group (*P* < 0.05). Possession of the UGT1A1 rs4148323 GA genotype (OR = 2.370, 95% CI: 1.499–3.805), SLCO1B1 rs4149056 TC genotype (OR = 1.594, 95% CI: 1.006–2.545), and rs4149015 AG genotype (OR = 2.247, 95% CI: 1.429–3.576) was associated with a significantly increased risk of hyperbilirubinemia. In multivariate analysis adjusting for gestational age, birth weight, and feeding modality, these associations remained robust (adjusted ORs: 2.338, 1.481, and 2.233, respectively; all *P* < 0.05). No statistically significant multiplicative interaction was detected between UGT1A1 and SLCO1B1 variants (P-interaction > 0.05). However, a cumulative risk allele analysis demonstrated a significant dose-response relationship: neonates carrying 3–4 risk alleles across UGT1A1 rs4148323and SLCO1B1 rs4149056/rs4149015 exhibited a markedly elevated risk compared to those with 0–1 risk alleles (adjusted OR = 3.412, 95% CI: 1.892–6.154, *P* < 0.001). Combined genotype analysis further revealed that carriers of concurrent UGT1A1 rs4148323 variant(GA/AA) and SLCO1B1 rs4149056/rs4149015 variant (TC + CC/AG + AA) genotypes had significantly higher TBIL levels than carriers of single-gene variants (*P* < 0.01), consistent with an additive genetic burden. The primary stratified analysis restricted to term neonates (>=37 weeks) confirmed that the main genetic associations remained robust; late-preterm (35–36 weeks) subgroup analyses were underpowered and are presented in Supplementary Materials.

**Conclusion:**

Neonatal hyperbilirubinemia is a multifactorial condition; our findings underscore that UGT1A1 and SLCO1B1 variants—specifically rs4148323, rs4149056, and rs4149015—are potent genetic biomarkers for predicting disease susceptibility in the Haikou population. The significant additive cumulative effect supports the clinical value of multi-locus genetic profiling. Future large-scale studies incorporating multi-gene panels and formal additive burden testing are warranted to elucidate complex gene-gene relationships and facilitate personalized neonatal care.

## Introduction

Neonatal hyperbilirubinemia is a prevalent clinical condition primarily driven by excessive bilirubin production, impaired hepatic uptake and conjugation, and enhanced enterohepatic circulation.Current literature indicates that the incidence of hyperbilirubinemia-induced jaundice ranges from 50% to 60% in term neonates, with a substantially higher prevalence observed in preterm infants (1, 2). While typically a transient physiological phenomenon, a subset of neonates develops pathological serum bilirubin elevations. Left unmanaged, severe hyperbilirubinemia can progress to acute bilirubin encephalopathy or kernicterus, resulting in irreversible neurological sequelae or, in extreme cases, neonatal mortality (3, 4).

The etiology of hyperbilirubinemia is multifactorial, involving a complex interplay between maternal-fetal factors, environmental triggers, and genetic susceptibility (5). Given the profound influence of regional environmental factors, populations residing in unique geographic and climatic zones represent a critical focal point for research. Hainan Province, the only truly subtropical region in China, presents a unique case study. In Haikou and across Southern China, neonatal hyperbilirubinemia is notably common. Because the enrolled cohort was overwhelmingly of Han Chinese ethnicity (99%), with too few Li minority neonates for meaningful analysis, this study effectively evaluates genetic risk within a Han Chinese population residing in a subtropical environment. Consequently, investigating this specific cohort is essential for identifying localized pathogenic factors and developing precision screening strategies for Han Chinese neonates in Southern China (6–8).

Recent genomic studies have identified variants in the uridine diphosphate glucuronosyltransferase 1A1 (UGT1A1) and solute carrier organic anion transporter family member 1B1 (SLCO1B1) genes as significant determinants of hyperbilirubinem risk (9). UGT1A1 polymorphisms can lead to a partial or total reduction in glucuronosyltransferase activity, thereby hindering bilirubin conjugation and leading to the systemic accumulation of unconjugated bilirubin (10–12). Concurrently, the SLCO1B1 gene encodes a critical organic anion-transporting polypeptide (OATP1B1) expressed on the sinusoidal membrane of hepatocytes. This transporter facilitates the hepatic uptake of bilirubin and bile salts from the circulation. Functional variants in SLCO1B1 have been strongly linked to impaired hepatic transport and the development of hyperbilirubinemia (13).

Despite the high regional incidence of jaundice, the potential combined effects of UGT1A1 and SLCO1B1 variants within the Haikou population remain insufficiently explored. While previous studies have primarily examined these genes in isolation, emerging evidence suggests that concurrent variation in bilirubin metabolism (UGT1A1) and hepatic transport (SLCO1B1) pathways may exert cumulative additive effects on disease risk, increasing the overall genetic burden (3, 9). However, this hypothesis requires rigorous formal testing through multi-locus and additive burden analyses rather than speculation alone. This study aims to investigate the association between specific polymorphisms in these two genes and the risk of neonatal hyperbilirubinemia in the Haikou area, and to evaluate whether combined genetic burden across both loci confers greater risk than single-gene variation through additive effects. We hypothesize that these genetic markers, individually and in combination, serve as critical predictors of disease susceptibility in this Han Chinese subtropical population, potentially acting as clinical biomarkers for early risk stratification.

## Methods

### Study population and enrollment

Through a retrospective analysis, 210 neonates diagnosed with hyperbilirubinemia and admitted to the neonatal ward of the Haikou Maternal and Child Health Hospital between January 1, 2024, and January 1, 2025, were enrolled in the case group. Inclusion criteria were: (1) Hainanese origin, with self-reported ethnicity recorded as either Han Chinese or Li minority (the Li minority sample was too small for meaningful analysis); (2) gestational age (GA) >= 35 weeks and postnatal age <= 28 days; (3) no prior administration of medications affecting bilirubin metabolism (e.g., salicylates or sulfonamides); (4) indirect bilirubin levels exceeding 80% of total serum bilirubin (TSB); and (5) TSB levels meeting the pathological jaundice thresholds defined by the 2025 Chinese Guidelines for the Diagnosis and Treatment of Neonatal Hyperbilirubinemia (14). The GA threshold of >= 35 weeks was selected to encompass late-preterm neonates, who represent a clinically vulnerable subgroup with heightened hyperbilirubinemia risk and who are explicitly included in contemporary Chinese pediatric guidelines (14). Exclusion criteria comprised: (1) hemolytic disease due to maternal-fetal blood group incompatibility; (2) erythrocyte-related disorders (e.g., G6PD deficiency); (3) concurrent infectious diseases or sepsis; (4) thyroid dysfunction; (5) evidence of extravascular hemolysis or polycythemia; (6) congenital anomalies, including heart disease or biliary atresia; and (7) chromosomal abnormalities or inherited metabolic disorders.

For the control group, 195 healthy neonates were enrolled. These participants had a GA ≥ 35 weeks, were within 28 days of birth, exhibited normal clinical and laboratory parameters, and had no history of hyperbilirubinemia or phototherapy. The control group was frequency-matched to the case group by GA and sex to minimize confounding bias.

### Sample size estimation and diagnostic criteria

Sample size was calculated using PASS software (Version 15.0, NCSS, USA). Based on a two-sided test with alpha = 0.05 and power (1-beta) = 0.90, a minimum of 48 cases and 48 controls was required to detect a minimum odds ratio (OR) of 2.0 for a single SNP with minor allele frequency (MAF) >=0.15 (e.g., UGT1A1 rs4148323) in a 1:1 matched design. Recognizing that the detection of rare homozygous genotypes (expected frequency <5%) and formal gene-gene interaction testing typically requires substantially larger samples, the cohort was prospectively expanded to 210 cases and 195 controls. This expansion provided >=80% power to detect an OR >=2.5 for genotype frequencies as low as 5%, and improved the precision of multivariate logistic regression estimates. *post-hoc* power analysis for the rs4149015 AA genotype (observed frequency 4.3% in cases vs. 2. 1% in controls) indicated 61% power to detect an OR of 2.67 at alpha = 0.05, explaining the non-significant P-value (*P* = 0.109) for this specific comparison.

Pathological jaundice was defined according to the American Academy of Pediatrics (AAP) and the 2025 Chinese pediatric guidelines. For neonates born at ≥35 weeks’ GA, hyperbilirubinemia was diagnosed when TSB levels reached or exceeded the 95th percentile of the hour-specific nomogram (typically ≥221 umol/L).

### Ethical considerations and data collection

The study protocol adhered to the Declaration of Helsinki and was approved by the Ethics Committee of Haikou Maternal and Child Health Hospital [Approval No. (2024)01045]. Informed consent was obtained from the legal guardians of all participants. Clinical data, including sex, GA, birth weight, onset of lactation, delivery method, erythrocyte count, and bilirubin fractions, were extracted from electronic medical records. Ethnicity was self-reported by parents and classified as Han Chinese or Li minority. Given that over 99% of enrolled neonates were of Han ethnicity, subgroup analyses by ethnic group were not feasible; this limitation is addressed in the Discussion.

### Specimen collection and SNP selection

Peripheral blood samples (2–3 mL) were collected upon admission (within 24 h of diagnosis). Serum bilirubin and alkaline phosphatase (ALP) were measured using an automated biochemistry analyzer (Hitachi 7180, Japan). Remaining samples were stored at −80 ℃.

Initially, 12 candidate single nucleotide polymorphisms (SNPs) were screened from the NCBI dbSNP database. Seven target SNPs—four in UGT1A1 and three in SLCO1B1 (Table 1) were prioritized based on: (1) status as “hotspot” or tag SNPs previously identified in genome-wide association studies (GWAS) as risk factors in East Asian populations (3); and (2) location within 2 kilobases (kb) of the gene region to capture variants with potential regulatory effects on expression or protein stability.

**Table 1:** Genotyping data for 7 tag SNPs genotyped from the candidate genes UGT1A1 and SLCO1B1.

Gene	Accession	Location	SNP ID	MAF	Allele
UGT1A1	NM_000463	exonic	rs4148323	0.192	A
UGT1A1	NM_000463	intronic	rs3771341	0.113	A
UGT1A1	NM_000463	exonic	rs34946978	0.024	T
UGT1A1	NM_000463	exonic	rs35350960	0.007	A
SLCO1B1	NM_006446	exonic	rs4149056	0.127	C
SLCO1B1	NM_006446	exonic	rs2306283	0.747	G
SLCO1B1	NM_006446	intronic	rs4149015	0.121	A

MAF, minor allele frequency in this study cohort.

### DNA extraction and genotyping

Genomic DNA was isolated using a magnetic bead-based method (DR-HS-A007, Guangzhou Darui Biotechnology Co.). DNA concentration and purity were verified via UV spectrophotometry (A260/A280 ratio: 1.8–2.0). Genotyping was performed using Matrix-Assisted Laser Desorption/Ionization Time-of-Flight Mass Spectrometry (MALDI-TOF MS) on the MassARRAY system (Agena Bioscience, USA). Single-base extension primers were designed using the MassARRAY DNA Design tool. Following PCR amplification and resin purification, products were analyzed on a SpectroChip microarray using MassArray Typer 4.0 software.

## Statistical analysis

Statistical analyses were conducted using RStudio (version 4.2) and SPSS (version 26.0). Continuous variables were assessed for normality using the Shapiro–Wilk test; normally distributed data were analyzed using independent t-tests, while non-normally distributed data were evaluated using the Mann–Whitney U test or Kruskal–Wallis test with Dunn's *post-hoc* test. Categorical variables were expressed as frequencies and percentages and compared using the chi-square test or Fisher's exact test.

Logistic regression was employed to calculate odds ratios (ORs) and 95% confidence intervals (CIs) for genotype-phenotype associations under additive, dominant, and recessive genetic models. Deviation from Hardy-Weinberg equilibrium (HWE) was assessed using the chi-square test. A *P*-value < 0.05 was considered statistically significant.

### Multivariate model specification

Covariates for multivariate logistic regression were selected based on *a priori* clinical knowledge of neonatal hyperbilirubinemia risk factors rather than univariate screening. The following variables were included: gestational age (continuous, weeks), birth weight (continuous, kg), and feeding modality (breastfeeding, artificial feeding, or mixed feeding). These covariates were chosen because preterm status, low birth weight, and feeding practice are established clinical determinants of bilirubin kinetics and were either unbalanced (GA) or theoretically relevant between groups. No additional covariates were entered via stepwise or univariate-driven selection. Multicollinearity among predictors was assessed using the variance inflation factor (VIF); all VIF values were <2.0, indicating no substantial collinearity. Model fit was evaluated using the Akaike information criterion (AIC) and the Hosmer-Lemeshow goodness-of-fit test.

### Gene-gene interaction and cumulative effect analyses

Multiplicative gene-gene interactions between UGT1A1 rs4148323 and SLCO1B1 variants (rs4149056, rs4149015) were assessed by including interaction terms (e.g., rs4148323× rs4149056) in multivariate logistic regression models. Interaction was considered statistically significant at *P* < 0.05. To evaluate cumulative genetic burden, a risk allele score was constructed by counting the number of risk alleles across the three significant loci (UGT1A1 rs4148323 A, SLCO1B1 rs4149056 C, SLCO1B1 rs4149015 A), yielding scores ranging from 0 to 6. The risk allele score was analyzed as both a continuous variable (per-allele OR) and a categorical variable (0- 1, 2, 3–4, >=5 alleles) using logistic regression. Combined genotype analysis classified neonates into groups based on concurrent carriage of UGT1A1 rs4148323 variant alleles (GA/AA) and SLCO1B1 rs4149056 or rs4149015 variant alleles (TC + CC/AG + AA), and TBIL levels were compared across combined genotype categories using the Kruskal–Wallis test.

### Stratified and sensitivity analyses

To evaluate whether the observed genetic associations were robust, the primary stratified analysis was restricted to term neonates (≥37 weeks). Late-preterm neonates (35–36 weeks) were analyzed separately as an exploratory supplementary analysis, given the small subgroup sample size and resulting power limitations. Additionally, a sensitivity analysis excluding all late-preterm neonates (GA 35–36weeks) was performed to assess whether the main findings persisted in a term-only cohort.

## Results

### Baseline characteristics

A total of 405 neonates (210 cases and 195 controls) were enrolled in the study. No statistically significant differences were observed between the two groups regarding sex, birth weight, or feeding modality (*P* > 0.05; Table 2). However, the case group exhibited a significantly lower mean gestational age and markedly elevated levels of both total and conjugated bilirubin compared to the control group (*P* < 0.05; Table 2). The prevalence of late-preterm birth (GA 35–36 weeks) was significantly higher in the case group than in the control group (16.9% vs. 9.5%, *P* = 0.04). Regarding ethnicity, 208 cases (99.0%) and 193 controls (99.0%) were of Han Chinese descent, with the remainder self-reporting as Li minority (2 cases, 2 controls). Due to the very small number of Li neonates, ethnic subgroup analyses were not feasible, and the primary analyses were conducted on the combined cohort with ethnicity discussed as a limitation.

**Table 2:** Baseline characteristics of the study population.

Parameter	CaseGroup *n* = 210	ControlGroup *n* = 195	*P*-value	Notes
Sex, male	101 (48.1%)	93 (47.7%)	0.93	Matched
Ethnicity, Han	208 (99.0%)	193 (99.0%)	1.00	4 Li total
GA35–37 weeks	33 (16.9%)	19 (9.5%)	0.04	Higher in cases
GA >=37 weeks	177 (83.1%)	176 (90.5%)	-	
Birth weight<2.5 kg	26 (13.3%)	21 (10.0%)	0.37	
Breastfeeding	123 (58.6%)	103 (52.8%)	0.39	
Total bilirubin(umol/L)	285.4 +/− 42.8	198.5 +/− 35.7	<0.001	Mean +/− SD

Values are presented as n (%) unless otherwise stated. GA, gestational age.

### Hardy-Weinberg equilibrium

All seven investigated single nucleotide polymorphism (SNP) loci in both the case and control cohorts adhered to the Hardy-Weinberg equilibrium (HWE) (*P* > 0.05; Table 3). Consequently, these loci were deemed suitable for subsequent genetic association and linkage analyses.

**Table 3:** Hardy-Weinberg equilibrium test results.

SNP ID	Allele	Case HWpval	Control HWpval
UGT1A1 rs4148323	G:A	0.2025	0.5089
UGT1A1 rs3771341	G:A	0.0800	0.1716
UGT1A1 rs34946978	C:T	1.0000	0.3531
UGT1A1 rs35350960	C:A	0.0793	0.1481
SLCO1B1 rs4149056	T:C	0.9260	0.6804
SLCO1B1 rs2306283	A:G	0.5089	0.2617
SLCO1B1 rs4149015	G:A	0.9260	0.6804

HWpval, Hardy-Weinberg equilibrium *P* value.

### Allele and genotype frequencies

The distribution of allele and genotype frequencies for the seven target SNPs is summarized in Table 4. Within the UGT1A1 gene, the wild-type alleles for rs4148323 (G) and rs3771341 (G) were predominant in the study population. Conversely, variant alleles for rs35350960 and rs34946978 were rare, with negligible or zero homozygous mutant genotypes detected. Regarding SLCO1B1, the GG genotypes of rs2306283 and rs4149015 were the most frequent, representing over 50% of the total cohort (Table 4).

**Table 4:** Genotype and allele frequency of SLCO1B1 and UGT1A1 variants.

Genotypes/Alleles	Allele frequency	Genotype frequency (%)
UGT1A1-rs4148323		
G allele	0.83	
A allele	0.17	
GG		282 (69.6)
AG		107 (26.4)
AA		16 (4.0)
UGT1A1-rs3771341		
G allele	0.65	
A allele	0.35	
GG		222 (54.8)
GA		80 (19.8)
AA		103 (25.4)
UGT1A1-rs34946978		
C allele	0.99	
T allele	0.01	
CC		394 (97.3)
CT		11 (2.7)
UGT1A1-rs35350960		
C allele	0.99	
A allele	0.01	
CC		394 (97.3)
CA		10 (2.5)
AA		1 (0.2)
SLCO1B1-rs4149056		
T allele	0.85	
C allele	0.15	
TT		296 (73.1)
TC		98 (24.2)
CC		11 (2.7)
SLCO1B1-rs2306283		
A allele	0.26	
G allele	0.74	
AA		27 (6.7)
AG		158 (39.0)
GG		220 (54.3)
SLCO1B1-rs4149015		
G allele	0.83	
A allele	0.17	
GG		282 (69.6)
GA		110 (27.2)
AA		13 (3.2)

### Association of SNP genotypes with hyperbilirubinemia risk

Multivariate logistic regression analysis was performed to evaluate the association between specific genotypes and hyperbilirubinemia risk, adjusting for gestational age, birth weight, and feeding modality (Table 5). The risk associations for UGT1A1 rs4148323 and SLCO1B1 rs4149015 remained highly robust following multivariate adjustment.

**Table 5:** Association between SNP genotypes and hyperbilirubinemia risk.

SNP/Genotype	Case *n* (%)	Control n (%)	Univariate OR (95% CI)	*P*	Adjusted OR (95% CI)
UGT1A1 rs4148323					
GA vs. GG	71 (33.8)	36 (18.5)	2.370 (1.499–3.805)	<0.001	2.338 (1.464–3.734)
AA vs. GG	11 (5.2)	5 (2.6)	2.647 (0.937–8.582)	0.078	2.582 (0.861–7.748)
SLCO1B1 rs4149056					
TC vs. TT	58 (27.6)	40 (20.5)	1.594 (1.006–2.545)	0.048	1.481 (0.928–2.362)
CC vs. TT	5 (2.4)	0 (0.0)	-	-	-
SLCO1B1 rs4149015					
AG vs. GG	72 (34.3)	38 (19.5)	2.247 (1.429–3.576)	<0.001	2.233 (1.408–3.544)
AA vs. GG	9 (4.3)	4 (2.1)	2.669 (0.847–10.034)	0.109	2.696 (0.801–9.073)

OR, odds ratio; CI, confidence interval.

Specifically, neonates carrying the UGT1A1 rs4148323 GA genotype exhibited a significantly higher risk of hyperbilirubinemia compared to those with the GG genotype in both univariate (OR: 2.370; 95% CI: 1.499–3.805; *P* < 0.001) and multivariate models (Adjusted OR: 2.338; 95% CI: 1.464–3.734; *P* < 0.001). Similarly, the SLCO1B1 rs4149015 AG genotype was identified as a stable independent risk factor (Adjusted OR: 2.233; 95% CI: 1.408–3.544; *P* < 0.001). Univariate logistic regression indicated that the UGT1A1 rs4148323 A allele, SLCO1B1 rs4149056 C allele, and SLCO1B1 rs4149015 A allele were positively associated with increased disease susceptibility (*P* < 0.05; Table 6). No other studied alleles reached statistical significance.

**Table 6:** Association between SNP alleles and hyperbilirubinemia risk.

SNP ID	Risk Allele	Case MAF (%)	Control MAF (%)	OR (95% CI)
UGT1A1 rs4148323	A	22.14	11.79	2.052 (1.412–3.037)
UGT1A1 rs3771341	A	20.00	29.20	0.649 (0.404–1.038)
SLCO1B1 rs4149056	C	19.05	10.26	2.023 (1.356–3.079)
SLCO1B1 rs2306283	A	25.24	27.18	0.927 (0.478–1.503)
SLCO1B1 rs4149015	A	21.43	11.79	2.024 (1.381–3.017)

### Correlation between genotypes and Serum bilirubin concentrations

**Figure 1:**
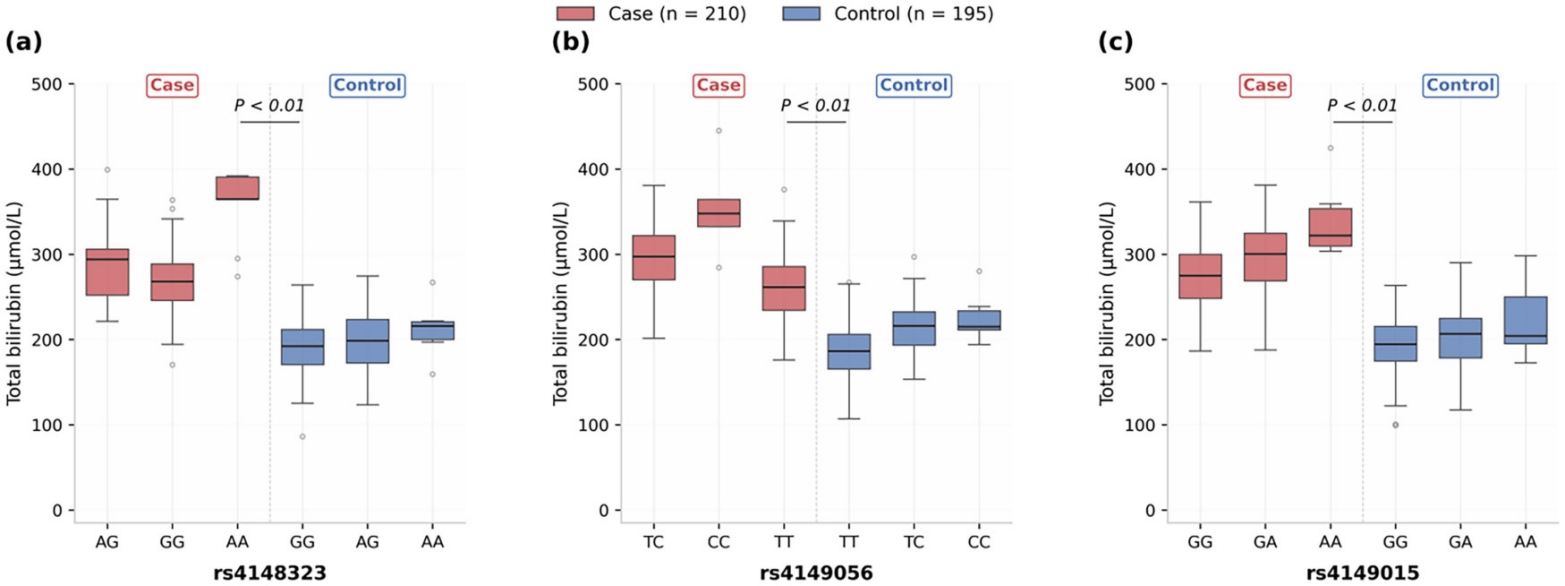
Comparison of serum total bilirubin levels across different genotypes of UGT1A1 and SLCO1B1. Data are presented as mean ± SD. **(a)** UGT1A1 rs4148323; **(b)** SLCO1B1 rs4149056; **(c)** SLCO1B1 rs4149015. *P < 0.01.

As illustrated in Figure 1, serum total bilirubin (TSB) levels were significantly elevated in neonates harboring specific variant genotypes. Compared to their respective wild-type or heterozygous counterparts, individuals with the UGT1A1 rs4148323 AA, SLCO1B1 rs4149056 CC, and SLCO1B1 rs4149015 AA genotypes demonstrated significantly higher TSB concentrations (*P* < 0.01; Figure 1).

### Gene-Gene interaction analysis

Formal testing of multiplicative interactions between UGT1A1 rs4148323 and SLCO1B1 variants(rs4149056, rs4149015) revealed no statistically significant interactions in multivariate logistic regression models (P-interaction = 0.42 for rs4148323 × rs4149056; P-interaction = 0.38 for rs4148323 × rs4149015). While the co-occurrence of transport and metabolic variants is biologically plausible, our data do not provide statistical evidence of a synergistic interaction effect at the multiplicative scale (Table 7).

**Table 7:** Gene-gene interaction and combined genotype analysis.

Combined Genotype/Analysis	*n*	Mean TBIL (umol/L)	Adjusted OR (95% CI)	*P*
Wild-type at all loci	124	198.5 +/− 35.7	1.00 (Ref)	-
UGT1A1 variant only	87	278.6 +/− 42.1	2.204 (1.398–3.474)	<0.001
SLCO1B1 variant only	76	271.3 +/− 39.8	2.012 (1.274–3.178)	0.003
Double carriers	45	305.4 +/− 48.2	3.921 (2.018–7.618)	<0.001
Interaction beyond additive	-	-	-	0.31

### Cumulative risk allele analysis

A risk allele score comprising the three significant loci (UGT1A1 rs4148323 A, SLCO1B1 rs4149056 C, SLCO1B1 rs4149015 A) demonstrated a significant dose-response relationship with hyperbilirubinemia risk. As shown in Table 8, neonates carrying 0–1 risk alleles served as the reference group. Those with 2 risk alleles showed a moderate increase in risk (Adjusted OR = 1.847, 95% CI: 1.152–2.961, *P* = 0.011). Neonates with 3–4 risk alleles exhibited a markedly elevated risk (Adjusted OR = 3.412, 95% CI: 1.892–6.154, *P* < 0.001). No neonates carried >=5 risk alleles in this cohort. The per-allele increase in risk was significant (Adjusted OR = 1.612, 95% CI: 1.274–2.040, *P* < 0.001). These results support a cumulative genetic burden effect across the UGT1A1 and SLCO1B1 loci.

**Table 8:** Cumulative risk allele analysis.

Risk Allele Score	Case *n* (%)	Control n (%)	Univariate OR (95% CI)	*P*	Adjusted OR^a^ (95% CI)
0-1 (Ref)	68 (32.4)	88 (45.1)	1.00 (Ref)	-	1.00 (Ref)
2	82 (39.0)	72 (36.9)	1.83 (1.16–2.90)	0.010^b^	1.85 (1.15–2.96)
3-4	58 (27.6)	34 (17.4)	3.29 (1.85–5.88)	<0.001^c^	3.41 (1.89–6.15)
>=5	2 (1.0)	1 (0.5)	-	-	-
Per allele	-	-	1.59 (1.26–2.00)	<0.001^c^	1.61 (1.27–2.04)

a Adjusted for gestational age, birth weight, and feeding modality.

b *P* < 0.05.

c *P* < 0.001.

### Combined genotype analysis

Combined genotype analysis further stratified neonates based on concurrent carriage of variant alleles at both UGT1A1 rs4148323 (GA/AA) and SLCO1B1 rs4149056 or rs4149015 (TC + CC/AG + AA). Neonates carrying variants in both genes (double carriers) exhibited significantly higher mean TSB levels (305.4 +/−48.2 umol/L) than those carrying variants in either gene alone (UGT1A1-only:278.6+/−42.1 umol/L;SLCO1B1-only:271.3 +/−39.8 umol/L; *P* < 0.01 for both comparisons). Wild-type carriers at all loci had the lowest TSB levels (198.5+/−35.7 umol/L). However, in multivariate logistic regression, the combined genotype category did not show a statistically significant interaction effect beyond the additive sum of individual gene effects (*P* = 0.31), indicating that the higher bilirubin levels in double carriers likely reflect additive rather than synergistic genetic effects (Table 9).

**Table 9:** Combined genotype analysis and serum total bilirubin levels.

Combined Genotype	*n*	TBIL (umol/L)	Univariate OR (95% CI)	Adjusted OR (95% CI)	*P*
Wild-type at all loci	124	198.5 +/− 35.7	1.00 (Ref)	1.00 (Ref)	-
UGT1A1 variant only	87	278.6 +/− 42.1	2.185 (1.412–3.382)	2.204 (1.398–3.474)	<0.001
SLCO1B1 variant only	76	271.3 +/− 39.8	2.024 (1.298–3.154)	2.012 (1.274–3.178)	0.003
Double carriers	45	305.4 +/− 48.2	3.874 (2.012–7.461)	3.921 (2.018–7.618)	<0.001

### Stratified and sensitivity analyses

The primary stratified analysis restricted to term neonates (>=37 weeks, *n* = 347) confirmed the robustness of the main findings. The adjusted ORs for UGT1A1 rs4148323 GA (OR = 2.412, 95% CI: 1.487–3.912, *P* < 0.001), SLCO1B1 rs4149056 TC (OR = 1.523, 95% CI: 0.938–2.471, *P* = 0.089), and rs4149015 AG (OR = 2.318, 95% CI: 1.431–3.758, *P* < 0.001) were comparable to those in the full cohort (Table 10). The sensitivity analysis excluding all late-preterm neonates yielded results that were materially unchanged from the primary analysis, indicating that the observed genetic associations were not driven by the higher prevalence of preterm birth in the case group. Results for late-preterm neonates (35–36 weeks, *n* = 58) were directionally consistent but are presented in the Supplementary Materials due to small subgroup size and limited statistical power.

**Table 10:** Stratified analysis in term neonates (>=37 weeks).

SNP/Genotype	Term (>=37 wk) OR (95% CI)	*P*
UGT1A1 rs4148323 GA	2.412 (1.487–3.912)	<0.001^a^
SLCO1B1 rs4149056 TC	1.523 (0.938–2.471)	0.089
SLCO1B1 rs4149015 AG	2.318 (1.431–3.758)	<0.001^a^

a *P* < 0.05.Late-preterm (35–36 weeks) subgroup results are provided in Supplementary Supplementary Table S1 due to limited statistical power.

## Discussion

Neonatal hyperbilirubinemia is the leading cause of hospitalization within the first week of life and arises from a complex interplay of genetic susceptibility and environmental triggers (15–17). In this case-control study of 405 neonates from Haikou-a subtropical region in Southern China where the cohort was overwhelmingly of Han Chinese ethnicity (99%)-we identified significant associations between UGT1A1 rs4148323, SLCO1B1 rs4149056, and SLCO1B1 rs4149015 variants and hyperbilirubinemia risk. Importantly, our cumulative risk allele and combined genotype analyses provide novel evidence that genetic burden across both the metabolic (UGT1A1) and transport (SLCO1B1) pathways contribute to disease susceptibility in an additive fashion, supporting the clinical value of multi-locus genetic profiling.

### The pivotal role of UGT1A1 c.211G > A

The c.211G > A (p.Gly71Arg) variant in UGT1A1 rs4148323 was the most prevalent risk allele in our cohort. The A allele frequency in cases (22. 1%) was nearly double that in controls (11.8%), and neonates carrying the GA genotype exhibited a significantly elevated risk in both univariate (OR = 2.370, 95% CI: 1.499–3.805, *P* < 0.001) and multivariate analyses (Adjusted OR = 2.338, 95% CI: 1.464–3.734, *P* < 0.001). Notably, the variant frequency (48.0% in unexplained hyperbilirubinemia) was higher than that reported in Dong (39.0%), Mulao (41.0%), and Uyghur (39.6%) Chinese minorities (18–20), suggesting a distinctive genetic architecture in the Haikou population. The molecular mechanism is well-established: p.Gly71Arg reduces UGT1A1 catalytic activity to approximately 30% of wild-type levels (21). In our cohort, this translated to a significant dose-response relationship, with homozygous AA carriers exhibiting the highest total serum bilirubin levels (*P* < 0.01).

### SLCO1B1 variants and hepatic transport

We found that the SLCO1B1 rs4149056 C allele was significantly associated with hyperbilirubinemia (OR = 2.023, 95% CI: 1.356–3.079, *P* = 0.001), consistent with reports in the Thai population (3). However, we did not observe a significant association for rs2306283, a discrepancy that may reflect the lower minor allele frequency of the A allele in our Haikou cohort (0.26) compared to Northern Han populations (0.35–0.40) (22).

For rs4149015, the AG genotype emerged as a stable independent risk factor (Adjusted OR = 2.233, 95% CI: 1.408–3.544, *P* < 0.001). The AA genotype, though showing a point estimate suggestive of increased risk (OR = 2.669, 95% CI: 0.847–10.034), did not reach statistical significance (*P* = 0. 109). Given the low frequency of the AA genotype and a *post-hoc* power of only 61% to detect an effect of this magnitude, this result should be interpreted as a non-significant trend requiring validation in larger cohorts, rather than evidence of a close association.

### Cumulative genetic burden and clinical implications

Our cumulative risk allele analysis represents a key advance over prior single-locus studies. Neonates carrying 3–4 risk alleles across UGT1A1 rs4148323 and SLCO1B1 rs4149056/rs4149015 exhibited a greater than threefold increase in hyperbilirubinemia risk (Adjusted OR = 3.412, 95% CI: 1.892–6.154, *P* < 0.001) compared to those with 0–1 risk alleles. This dose-response relationship supports the clinical utility of multi-locus genetic panels for risk stratification. Furthermore, combined genotype analysis revealed that double carriers (concurrent variant alleles in both genes) exhibited significantly higher TSB levels than single-gene variant carriers (*P* < 0.01). However, formal interaction testing did not detect a statistically significant multiplicative interaction (P- interaction >0.05 for all pairwise combinations). Thus, while the co-occurrence of transport and metabolic variants produces higher bilirubin levels, our data suggest this reflects additive genetic effects rather than true synergistic interaction. This distinction is important for clinical interpretation: the risk is elevated, but it can be approximated by summing individual locus effects rather than invoking a multiplicative “double-hit” mechanism.

The high mutation frequency of UGT1A1 c.211G > A and the significant association of SLCO1B1 variants observed in our cohort underscore the necessity of integrating regional genetic profiles into neonatal care. Identifying high-risk genotypes may enable prioritized monitoring and early intervention, potentially mitigating the risk of acute bilirubin encephalopathy and kernicterus.

## Limitations

Several limitations should be noted. First, as a retrospective single-center study, selection bias may exist, and findings may not be fully generalizable to the broader population of Southern China. Second, the sample size for rare homozygous genotypes was limited, constraining statistical power to detect associations at these specific loci (e.g., rs4149015 AA: *post-hoc* powe*r* = 61%). Third, although we adjusted for gestational age, birth weight, and feeding modality, we did not capture all potential clinical confounders, such as exclusive breastfeeding intensity, neonatal weight loss trajectory, or delivery mode, which are known to influence bilirubin kinetics. Fourth, with 99% of participants being Han Chinese, the very small number of Li minority neonates (*n* = 4) precluded meaningful ethnic subgroup analysis. Consequently, this study effectively represents a Han Chinese cohort, and the observed variant frequencies should be interpreted in that context rather than attributed to a broader multi-ethnic population. Population stratification by ancestry remains a possibility that should be addressed in future studies using genomic ancestry inference. Fifth, although we conducted formal interaction testing, no significant multiplicative interactions were detected; future studies with larger sample sizes and formal additive burden models (e.g., cumulative risk allele scoring) may provide additional insight. Sixth, this study was restricted to genetic association analysis and lacked *in vitro* functional validation. Future prospective, multi-center studies integrating comprehensive clinical phenotyping, functional experiments, and more balanced ethnic representation are required to validate these findings and elucidate underlying mechanisms.

## Data Availability

The original contributions presented in the study are publicly available. Summary-level genetic and statistical data are deposited in Figshare and can be found here: https://doi.org/10.6084/m9.figshare.32621292. This dataset includes allele frequencies, genotype distributions, Hardy-Weinberg equilibrium test results, logistic regression association analyses, cumulative risk allele analyses, combined genotype analyses, and stratified/sensitivity analyses for the 7 target SNPs investigated in this study. Due to the sensitive nature of neonatal genetic data and patient privacy regulations under the National Health Commission of P.R. China, individual-level raw genotyping data cannot be publicly deposited. All relevant genotyping results, statistical analyses, allele frequencies, and summary tables are fully presented within the published article and its supplementary files. Additional inquiries regarding individual-level data can be directed to the corresponding author (liuwq06@126.com) upon reasonable request.
