# Supplementary material for: Variants in UGT1A1 and SLCO1B1 increase the risk of neonatal hyperbilirubinemia: a case-control study in subtropical China
Source: Front Pediatr. 2026 Jun 24;14:1843414. doi: 10.3389/fped.2026.1843414 (PMC13341593; doi:10.3389/fped.2026.1843414)
Supplement: Supplementary file 1 [file Datasheet1.docx]

**Supplementary Table S1**

*Late-Preterm (35-36 weeks) Subgroup Analysis: Association between UGT1A1 and SLCO1B1 Variants and Neonatal Hyperbilirubinemia Risk*

*Note: This supplementary table presents exploratory analyses of the late-preterm neonate subgroup (gestational age 35-36 weeks, n=58). Due to the small sample size (33 cases, 25 controls), these analyses are underpowered and results should be interpreted with caution. Findings were directionally consistent with the main analysis.*

**Table S1A. Baseline Characteristics of Late-Preterm Neonates (35-36 weeks)**

| **Parameter** | **Case (n=33)** | **Control (n=25)** | **P-value** | **Notes** |
| --- | --- | --- | --- | --- |
| Sex, male | 16 (48.5%) | 12 (48.0%) | 0.97 | Matched |
| Gestational age (weeks), mean +/- SD | 35.6 +/- 0.5 | 35.7 +/- 0.5 | 0.42 |  |
| Birth weight <2.5 kg | 8 (24.2%) | 5 (20.0%) | 0.71 |  |
| Breastfeeding | 19 (57.6%) | 14 (56.0%) | 0.90 |  |
| Total bilirubin (umol/L), mean +/- SD | 312.4 +/- 48.6 | 205.3 +/- 38.2 | <0.001 | Mean +/- SD |
| Indirect bilirubin (umol/L), mean +/- SD | 285.7 +/- 45.3 | 182.6 +/- 35.8 | <0.001 | Mean +/- SD |

**Table S1B. Genotype and Allele Frequencies in Late-Preterm Neonates**

| **SNP / Genotype** | **Case n (%)** | **Control n (%)** | **OR (95% CI)** | **P-value** |
| --- | --- | --- | --- | --- |
| **UGT1A1 rs4148323** |  |  |  |  |
| GG | 19 (57.6%) | 18 (72.0%) | 1.00 (Ref) |  |
| GA | 11 (33.3%) | 6 (24.0%) | 1.737 (0.512-5.893) | 0.376 |
| AA | 3 (9.1%) | 1 (4.0%) | 2.842 (0.267-30.28) | 0.385d |
| **SLCO1B1 rs4149056** |  |  |  |  |
| TT | 22 (66.7%) | 18 (72.0%) | 1.00 (Ref) |  |
| TC | 9 (27.3%) | 6 (24.0%) | 1.227 (0.362-4.158) | 0.739 |
| CC | 2 (6.1%) | 1 (4.0%) | 1.636 (0.135-19.84) | 0.694d |
| **SLCO1B1 rs4149015** |  |  |  |  |
| GG | 20 (60.6%) | 19 (76.0%) | 1.00 (Ref) |  |
| GA | 11 (33.3%) | 5 (20.0%) | 2.091 (0.598-7.314) | 0.244 |
| AA | 2 (6.1%) | 1 (4.0%) | 1.900 (0.156-23.14) | 0.620d |

**Table S1C. Association between SNP Genotypes and Hyperbilirubinemia Risk in Late-Preterm Neonates**

| **SNP / Genotype** | **Univariate OR (95% CI)** | **P** | **Adjusted ORb (95% CI)** | **P** |
| --- | --- | --- | --- | --- |
| **UGT1A1 rs4148323** |  |  |  |  |
| GA vs GG | 1.737 (0.512-5.893) | 0.376 | 1.692 (0.481-5.953) | 0.412 |
| AA vs GG | 2.842 (0.267-30.28) | 0.385 | 2.756 (0.251-30.24) | 0.401 |
| **SLCO1B1 rs4149056** |  |  |  |  |
| TC vs TT | 1.227 (0.362-4.158) | 0.739 | 1.185 (0.338-4.154) | 0.789 |
| CC vs TT | 1.636 (0.135-19.84) | 0.694 | 1.582 (0.128-19.56) | 0.712 |
| **SLCO1B1 rs4149015** |  |  |  |  |
| AG vs GG | 2.091 (0.598-7.314) | 0.244 | 2.024 (0.558-7.342) | 0.274 |
| AA vs GG | 1.900 (0.156-23.14) | 0.620 | 1.834 (0.148-22.74) | 0.638 |

**Table S1D. Hardy-Weinberg Equilibrium Test Results for Late-Preterm Neonates**

| **SNP ID** | **Alleles** | **Case HWE P-value** | **Control HWE P-value** |
| --- | --- | --- | --- |
| UGT1A1 rs4148323 | G:A | 0.5821 | 0.4238 |
| UGT1A1 rs3771341 | G:A | 0.3124 | 0.2156 |
| UGT1A1 rs34946978 | C:T | 1.0000 | 1.0000 |
| UGT1A1 rs35350960 | C:A | 1.0000 | 1.0000 |
| SLCO1B1 rs4149056 | T:C | 0.6874 | 0.5123 |
| SLCO1B1 rs2306283 | A:G | 0.4521 | 0.3876 |
| SLCO1B1 rs4149015 | G:A | 0.7234 | 0.5342 |

Abbreviations: GA, gestational age; OR, odds ratio; CI, confidence interval; HWE, Hardy-Weinberg equilibrium; SNP, single nucleotide polymorphism; TBIL, total bilirubin.

a Values are presented as n (%) unless otherwise stated.

b Adjusted for birth weight and feeding modality (gestational age excluded due to narrow range in this subgroup).

c P<0.05 indicates statistical significance.

d Fisher's exact test used when expected cell count <5.

e Due to small sample size and limited statistical power, these results are provided for exploratory purposes only.
